# Repeated cyclone events reveal potential causes of sociality in coral-dwelling *Gobiodon* fishes

**DOI:** 10.1371/journal.pone.0202407

**Published:** 2018-09-05

**Authors:** Martin L. Hing, O. Selma Klanten, Mark Dowton, Kylie R. Brown, Marian Y. L. Wong

**Affiliations:** 1 Centre for Sustainable Ecosystems Solutions, School of Biological Sciences, University of Wollongong, Wollongong, Australia; 2 Fish Ecology Laboratory, School of Life Sciences, University of Technology Sydney, Sydney, Australia; 3 Centre for Medical and Molecular Bioscience, School of Biological Sciences, University of Wollongong, Wollongong, Australia; 4 Independent Researcher, Sanctuary Point, NSW, Australia; Department of Agriculture and Water Resources, AUSTRALIA

## Abstract

Social organization is a key factor influencing a species’ foraging and reproduction, which may ultimately affect their survival and ability to recover from catastrophic disturbance. Severe weather events such as cyclones can have devastating impacts to the physical structure of coral reefs and on the abundance and distribution of its faunal communities. Despite the importance of social organization to a species’ survival, relatively little is known about how major disturbances such as tropical cyclones may affect social structures or how different social strategies affect a species’ ability to cope with disturbance. We sampled group sizes and coral sizes of group-forming and pair-forming species of the Gobiid genus *Gobiodon* at Lizard Island, Great Barrier Reef, Australia, before and after two successive category 4 tropical cyclones. Group sizes of group-forming species decreased after each cyclone, but showed signs of recovery four months after the first cyclone. A similar increase in group sizes was not evident in group-forming species after the second cyclone. There was no change in mean pair-forming group size after either cyclone. Coral sizes inhabited by both group- and pair-forming species decreased throughout the study, meaning that group-forming species were forced to occupy smaller corals on average than before cyclone activity. This may reduce their capacity to maintain larger group sizes through multiple processes. We discuss these patterns in light of two non-exclusive hypotheses regarding the drivers of sociality in *Gobiodon*, suggesting that benefits of philopatry with regards to habitat quality may underpin the formation of social groups in this genus.

## Introduction

Social organization is an important determinant of a species’ survival [1], foraging efficiency [2] and ability to reproduce successfully [3], factors which ultimately affect their potential to recover from disturbances. Social structures may be as simple as monogamous pairing or as complex as a eusocial colony with division of labour and non-reproductive castes. Social organization may be influenced by broad ecological [4] or life-history factors [5], within-group social interactions [6] or genetic relatedness [7] and even individual variation in physiology [8], neurophysiology and genetics [9]. Each social structure provides benefits to its constituents, but often at a cost to their reproduction or access to some other resource [10–12]. That is to say, there are trade-offs associated with different social structures that individuals must consider.

Group living is thought to have evolved in many lineages as a response to genetic (kinship) and environmental factors [10–12]. With respect to environmental factors, many hypotheses point toward variability in ecological factors as influencing the evolution of sociality [4, 13]. Hypotheses such as the benefits of philopatry [14–16] and ecological constraints models [4, 16–19] examine the idea that ecological factors, such as habitat quality (e.g. habitat size, resource availability, defence) or the availability of suitable breeding territory (respectively), influence the decision of subordinates to either disperse from their habitat or remain within a group.

These two hypotheses are often viewed as two sides of the same coin as they both look at aspects of ecology to explain social evolution and maintenance [20]. The benefits of philopatry hypothesis focuses on the benefits conferred from residing in a high-quality habitat (e.g. inheritance of breeding status [21], increased fitness [22]). High-quality habitat is typically colonized rapidly [13]. An individual living on low-quality habitat may therefore increase its fitness by moving to a high-quality habitat as a subordinate [13]. However, this benefit must be traded off against the associated costs (e.g. delayed reproduction, risk of movement). In contrast, the ecological constraints hypothesis concentrates on factors of ecology that may restrict subordinate individuals already residing in a group from dispersing (e.g. habitat saturation [23], predation risk [24]). These two hypotheses are not mutually exclusive and often operate alongside other effects (e.g. kinship, life-history). However, the question of which combination of effects best describes social group formation and maintenance is still of interest as each one emphasizes different costs and benefits [25].

While these hypotheses have been well studied in terrestrial organisms, they have only recently been tested in marine environments [20, 26–28]. Of the marine taxa tested so far, habitat-specialist coral-reef fishes are emerging as a useful model species to study theories of social evolution and maintenance [20, 27, 28] and have shown similar responses to habitat manipulation as terrestrial species (e.g. [23]). Many social fishes have a pelagic larval phase which suggests low levels of kinship within groups, reducing the potential confounding factor of relatedness (e.g. [20, 29, 30] but see [31]). Given the apparent influence of ecological factors on the formation and maintenance of social groups, we would expect that disturbances capable of altering a species’ habitat, such as severe weather events, would have a strong impact on social organization [23, 32].

Many species of coral-reef fishes, especially habitat-specialists, can be found in social groups [33, 34–36]. The size of these social groups is often related directly or indirectly to the size of the habitat in which they reside [36–39]. Complex social structures such as size-based dominance hierarchies, in which the largest dominants breed and smaller subordinates are reproductively suppressed, have been documented in these groups [23, 38]. Further, they are known to exhibit sequential hermaphroditism or bi-directional sex-change [38, 40, 41]. In such systems, the loss of a breeding individual results in the next subordinate in the queue taking its place [6]. This social organization may provide a level of redundancy which could help a social species recover quickly following a major disturbance. For example, Rubenstein [42] argued that cooperative breeding could be a bet hedging strategy in variable environments as it may buffer variance in fecundity between years. Duffy and Macdonald [26] also found that eusociality conferred advantages to sponge-dwelling shrimps allowing them to occupy a greater number of host sponge species and more sponges overall than less social sister species. This finding, combined with research on host specialization and extinction by Munday [43], could imply that more social species face lower extinction risk following a disturbance because their sociality allows them to monopolize a greater host range. However, Courchamp et al. [44] found that obligate cooperative breeders were more at risk of group extinction because of their reliance on subordinates to reproduce and survive. These studies show that while complex social structures may provide advantages allowing species to survive a severe disturbance and to re-colonize afterwards, they may also result in localized extinctions. Further research into the effects of ecological disturbance on social organization and how varying social systems, such as pair- or group-forming, are able to cope with disturbance are clearly required.

Extreme climatic events such as tropical cyclones are known to have devastating impacts on the physical structure of coral reefs [45–48]. The effects on fish and invertebrate communities which depend on the coral structure for food and shelter are likewise devastating [45, 49–51]. The destructive forces of cyclones can have a strong influence on the re-distribution of species and their relative abundances following the event [52]. However, relatively little is known about the impacts that cyclones may have on the social organization of coral-reef inhabitants and whether social organization may mediate disturbance-induced population trends in species with different social structures. Given the importance of social organization for factors such as reproduction [3], foraging efficiency [2] and ultimately the ability to recover from a major disturbance, it is plausible that destructive events such as tropical cyclones may have a detectable effect on a species’ social organization.

We evaluated the effects of cyclones on the social organization of coral gobies of the genus *Gobiodon*. These species are small (3–4 cm) microbenthic [36] habitat-specialist fishes that live within the structures of branching and plate-forming acroporid and pocilloporid corals [53, 54]. These fishes are highly site attached once settled, but have been shown to move between corals [41]. *Gobiodon* spp. display a wide variety of social phenotypes from pair-forming (PF) species to group-forming (GF) species that typically live in groups ranging from 3 to 12 individuals [55]. Social groups usually consist of two breeding individuals and one or more non-breeding subordinates which form a size-based hierarchy and queue for a breeding position. However there is some evidence to support multiple breeding individuals in larger group sizes for some species [55].

In this study, we investigated how extreme climatic events influence the social organization of colonies of *Gobiodon* fishes and discuss how these effects may impact their survival. Opportunistic investigations of such disturbances (extreme climatic events) are important for theory development as they can test well developed theory under extreme conditions [56]. Specifically, we examined the effects of two successive category 4 cyclones that impacted the Great Barrier Reef, on the group size (social structure) and coral size (ecological factor) of GF and PF species of *Gobiodon*. As habitat patch size is known to be related to mean group size in some species, smaller corals should be less capable of supporting larger groups [55]. Therefore, we expected that physical damage caused by the cyclones would result in smaller corals, and that as coral size decreased, so too would mean group size of both GF and PF coral gobies. We also expected that advantages conferred from sociality would help GF species to recover from these disturbances [26, 43].

Additionally, we used the occurrence of these cyclones as a ‘natural experiment’ to examine the related effects of ecological constraints and benefits of philopatry on the formation of social groups in the GF species. Munday [57] demonstrated that coexistence between two species of *Gobiodon* occurred through a competitive lottery, meaning that whichever species colonized a particular coral was able to hold that territory. Our own observations show that while coral gobies do show distinct preferences for certain species of coral, they can and will colonize a wide range of species. It is therefore likely that *Gobiodon* species will colonize any available habitat following a severe disturbance. If ecological constraints (lack of available habitat) were responsible for the formation of social groups, we would expect coral vacancy to be very low as gobies would preferentially colonize vacant habitat over residing as a subordinate in a group. That is, subordinates should disperse to seek independent breeding opportunities if there is suitable vacant habitat. In contrast, if benefits of philopatry were driving group living, we would expect greater coral vacancy as the GF species would vacate lower-quality corals in favor of taking up residence as a subordinate in higher-quality corals. While we do not fully understand what constitutes high- or low quality habitat in these species, we consider coral size to be a reasonable proxy of habitat quality as Kuwamura et al. [58] and Hobbs and Munday [59] demonstrated that growth, survival and reproductive success increased in larger habitats for other species of coral associated fishes.

## Materials and methods

### Ethics statement

This research was conducted under research permits issued by the Great Barrier Reef Marine Park Authority (G13/36197.1 and G15/37533.1) and with the approval of the University of Wollongong Animal Ethics Committee (AE14-04).

### Study area and survey sites

**Fig 1:**
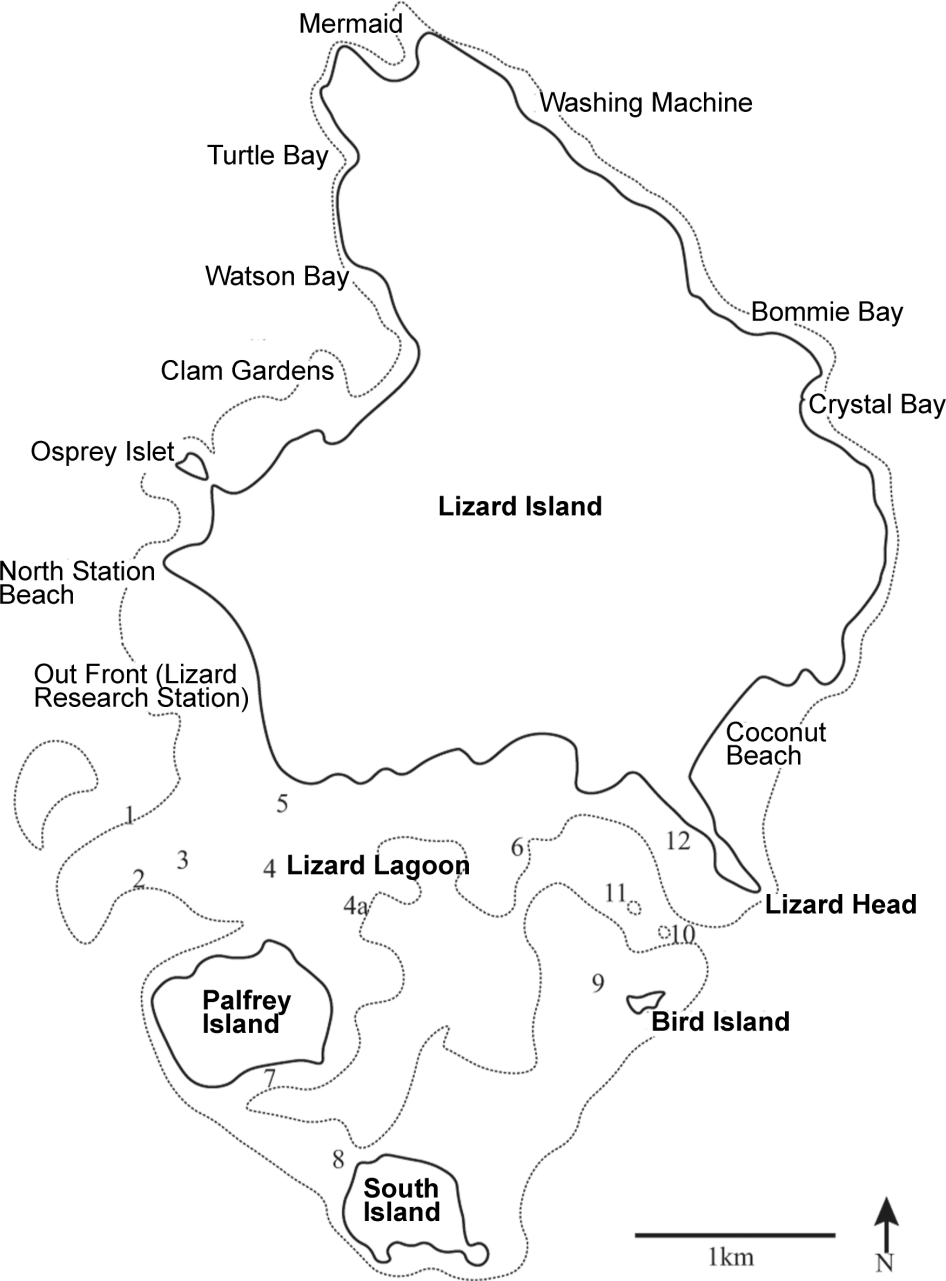
Map of the survey sites. Dotted light grey line is the outline of reef areas around Lizard Island. All study sites are indicated on map (regular font), specific reefs in the Lizard Island lagoon are numbered: Big Vickey’s Reef (1); Vickey’s Reef (2); Horse Shoe Reef (3); Palfrey Reef (4–4a); Loomis Reef (5); Trawler (6); Picnic Beach (7); Ghost Beach (8); Bird Island Reef (9); Entrance Bommie (10); Bird Bommie (11); Lizard Head Reef (12).

The study took place at Lizard Island, Great Barrier Reef, Queensland, Australia (14° 40.729’ S, 145° 26.907’ E) (Fig 1) between 2014 and 2016. Twenty three sites were surveyed in total over four survey times, eleven of which were located within the sheltered lagoon. The remaining twelve sites were located on the fringing reefs around Lizard Island. As this study was designed to examine how sociality of *Gobiodon* spp. varied over successive impacts at Lizard Island as a whole, we did not assess variation in sociality at smaller spatial scales (e.g. sites). As such, survey sites were chosen to give reasonable coverage of the reefs at Lizard Island. Not all sites were assessed during each survey time as several sites were scoured down to bare rock after each cyclone. These sites were not surveyed as our interest was in the surviving goby colonies (see S1 Data for the range of sites covered at each survey time). The number of sites visited during each survey time was 15, 14, 11, 17 respectively. All measurements were made on scuba at depths ranging from less than one meter to five meters.

### Cyclone activity and sampling periods

**Fig 2:**
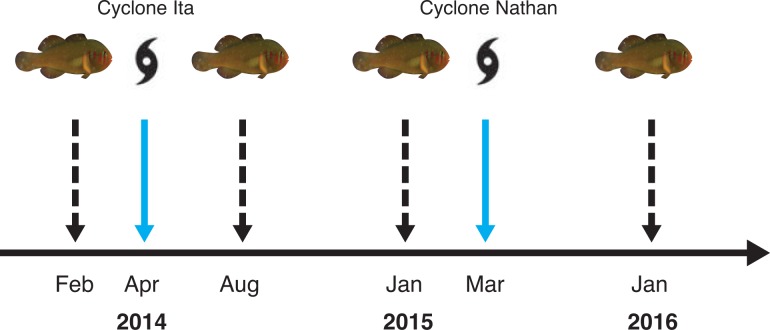
Timeline of data collection. Timeline shows year and month of data collection (fish, dashed black arrow) and cyclone activity (cyclone, blue arrow). In total, data on group size, coral size and proportion of corals occupied were collected at 4 time points for 13 species of *Gobiodon*.

Two cyclones impacted the study site in consecutive years. Cyclone Ita impacted Lizard Island in April 2014 as a category 4 system and cyclone Nathan in March 2015, also as a category 4 system. Both cyclones caused substantial damage to the fringing and lagoonal reefs including greatly reduced coral cover and associated changes in reef fish diversity and abundance [60–63]. We conducted surveys on coral sizes and group sizes of 13 *Gobiodon* spp. during February and March 2014 (1 month prior to cyclone Ita), August and September 2014 (4 months after cyclone Ita), January and February 2015 (1 month prior to cyclone Nathan and 9 months after cyclone Ita) and January and February 2016 (10 months after cyclone Nathan) (Fig 2). These repeated surveys provided us with a broad overview of the effects that multiple disturbances had on the social organization of coral gobies.

### Survey methods

Two types of transects were deployed over the four surveys. For this study however, we did not attempt to assess any spatial patterns between sites. Transects were only used as a guide to locate corals. Haphazardly placed 30 m line transects were used to locate corals one meter either side during the first and fourth survey times. Cross transects (two 4 m x 1 m belt transects laid in a cross, designed to measure the community around a focal colony) were used during the first (Palfrey reef only; Fig 1, sites 4 and 4a), second, third and fourth survey times. Line transects were placed roughly parallel to each other and separated by at least 10 m and cross transects were placed at least 8 m (twice the length of the transect on either axis) from each other to ensure that any given coral was not measured twice during the survey period. As coral gobies show strong preferences for certain species of branching and plate forming (mostly) Acroporid corals [35, 53, 64], only these species of corals were counted on the transects. In total, 23 species of coral were surveyed for goby occupancy (S1 Data). Each coral’s living part was measured along three axes (length (L) width (W) and height (H)) and the simple average diameter calculated as (L + W + H)/3. Simple average diameter was used in this study (as opposed to geometric mean diameter (L x W x H)^1/3^) as it provides a better representation of the major axis of the coral [58]. All goby supporting corals occurring on the transects were measured and searched for gobies. The number of adult gobies living within each coral head was counted by visual inspection using a torch. Adults could be easily distinguished from juveniles by their distinct coloration and markings. While the number of juveniles (if present) was recorded for each coral, they were not included in the group size observations as juveniles had been observed moving between multiple corals during each survey (Hing pers. obs.). Additionally, juvenile abundance was extremely low during all surveys and there was no difference in abundance for either PF or GF species during any survey time (S2 Fig). In contrast, adults displayed remarkable coral-host fidelity, even tolerating extreme hypoxia and severe coral bleaching [65, 66].

The number of transects at each site varied depending on the size of the reef. The number of transects conducted at each site also varied from year to year depending on the perceived abundance of suitable corals for habitation, and ranged from 1 to 44 transects. In total, the number of transects placed around Lizard Island during each survey time was 56, 141, 109 and 140 for the February 2014, August 2014, January 2015 and January 2016 surveys respectively. The methods of measuring goby group sizes and coral sizes (described in detail below) remained exactly the same regardless of the different number and size of transects that were used throughout the study. There was a significant difference in coral size measured between the two transect types, however this was most likely a site effect as line transects were used extensively on the fringing reefs in January 2016, after both cyclones. Corals at these sites sustained heavy damage and were therefore smaller on average. We therefore pooled the data from both types of transect and included site as a random effect in the statistical models.

### Sociality in *Gobiodon*

We documented 15 species of *Gobiodon* at Lizard Island during the present study (Table 1). However, two species (sp. A and sp. D) were excluded from later analyses as they were uncommon at the study site. The remaining species displayed a range of sociality ranging from solitary individuals to pairs and groups reaching up to 21 individuals. From here on we will use the term “group” to refer to any colony with a group size of three or more. We used a sociality index formulated by Avilés and Harwood [67] to categorize each species as either GF or PF:

$$Sociality=\frac{\left(\frac{A_{d}}{A_{a}})+(\frac{N_{g}}{N_{g}+N_{p}+N_{i}})+(\frac{I_{n}}{I_{r}+I_{n}}\right)}{3}$$ (1)

Where A_d_ = age at dispersal, A_a_ = age when adulthood is reached, N_g_ = number of groups, N_p_ = number of pairs, N_i_ = number of solitary adults, I_r_ = number of reproducing adults and I_n_ = number of non-reproducing (subordinate) adults. The three components in the numerator of Eq 1 represent the proportion of a species’ life-cycle spent in a group, the proportion of groups in the population and the proportion of subordinates in the population (respectively).

**Table 1:** List of *Gobiodon* spp. and sociality categorization.

Species	Individuals	Groups	Sociality index	Categorization
*G*. *axillaris*	15	9	0.33	Pair
*G*. *brochus*	70	35	0.36	Pair
*G*. *ceramensis*	36	20	0.36	Pair
*G*. *erythrospilus*	138	69	0.41	Pair
*G*. *histrio*	79	43	0.43	Pair
*G*. *oculolineatus*	59	30	0.39	Pair
*G*. *okinawae*	33	19	0.46	Pair
*G*. *quinquestrigatus*	114	59	0.38	Pair
*G*. *acicularis*	48	17	0.56	Group
*G*. *citrinus*	37	9	0.63	Group
*G*. *fuscoruber*^a^	142	51	0.57	Group
*G*. *rivulatus*	145	45	0.65	Group
Unknown species	28	8	0.63	Group

*Gobiodon* spp. observed at Lizard Island with their social index. The number of individuals and groups of each species recorded during the February 2014 survey are provided. Species were categorized as group-forming (below dotted line) if their social index was greater than 0.5. Otherwise they were categorized as pair-forming (above dotted line).

^a^ *G*. *fuscoruber* is synonymous with *G*. *unicolor* [76]

Using this equation, we calculated a sociality index for each *Gobiodon* spp., making some necessary but biologically relevant assumptions. Once coral gobies settle onto a coral as juveniles, they are not known to move frequently unless forcefully evicted from the coral [6, 68]. Although we do not have a precise estimate of the age at settlement for each species, Brothers et al. [69] estimated the larval life of three species of *Gobiodon* ranging from 22 to 41 days. Given that *Gobiodon* spp. live in the order of years [29, 70], we assume that each species spends the majority of its life-cycle in a single coral. We therefore set the maximum proportion of the life-cycle spent in the group ($\frac{A_{d}}{A_{a}}$) as 1 for each species. While there may be natural variation in this parameter, this assumption is biologically realistic and enables us to make relative comparisons between species primarily based on the remaining two factors in Eq 1. The last two components of the index were calculated as per Eq 1.

Having calculated the sociality indices, species were categorized as GF if their sociality index was greater than 0.5 and remaining species with sociality indices less than 0.5 were categorized as PF (Table 1). The index value of 0.5 was defined as the cut-off value between PF and GF species because it lies directly in the middle of the observed index range where there was a natural split in the data (S1 Fig). It should be noted however that “PF” species were sometimes observed in groups (i.e. 3 or more individuals) and “GF” species were sometimes observed in pairs or as singles. The terminology used here therefore indicates the tendency of particular species to form either groups or pairs. Importantly, calculations of sociality indices and subsequent categorization was based on data from surveys obtained before any recent cyclone activity (February 2014). We acknowledge that these reefs have been subjected to Crown of Thorns Starfish (COTS) outbreaks in the past. Our measure of sociality may therefore vary from sociality recorded at other locations. Unfortunately, COTS outbreaks are a relatively frequent occurrence on the Great Barrier Reef and we therefore consider our measure of sociality to be representative of the ‘normal’ social organization of the species in question.

### Group size

To assess the effect of cyclone activity on social organization, we used a generalized linear mixed model with a zero-truncated negative binomial distribution to analyze the effects of sociality and survey time and their interaction on the group size of coral gobies. The zero truncated distribution was used as it does not allow predictions of group size less than one. A negative binomial distribution was used to account for over-dispersion which rendered an initially employed zero-truncated Poisson model unsuitable. The model contained survey time (Feb-14, Aug-14, Jan-15 and Jan-16), social organization (PF or GF) and the interaction between these factors as fixed effects. Site, coral species and goby species were included as crossed random effects. Root mean square error (RMSE) was used to assess model performance. RMSE is a measure of the overall agreement between model predictions and the observed data and is measured in the same unit as the response variable. Generalized linear mixed models were conducted in R using the glmmADMB package [71, 72] and pairwise comparisons conducted with the emmeans package [73]. Figures were produced using the ggplot2 package [74].

### Coral size and abundance of empty corals

To investigate changes in coral sizes for PF and GF species over the four survey times, we tested the relationship between social organization, survey time and coral size. We used a generalized linear mixed model with survey time, social organization and their interaction as fixed effects and site, goby species and coral species as crossed random effects. A gamma distribution was used to account for positive skew and heteroscedasticy in the data and because it gave a better fit than models conducted with log-normal distributions. RMSE was used to assess model performance.

To test the hypothesis that subordinates (i.e. non-reproducing individuals) in colonies of GF species might be constrained by a lack of available habitat, we also assessed whether the mean number of empty corals on a transect was different for transects with or without groups of GF species. We used a generalized linear model for this analysis with the number of empty corals as the dependent variable and survey time and transect type (with or without groups of GF species) as independent variables. The model was run with a zero-inflated negative binomial distribution to handle the large number of zero counts and this produced a better model than a zero-inflated Poisson model when compared with Akaike Information Criterion (AIC). The model was conducted using the R package glmmADMB [71, 72].

### Proportion of inhabited corals and probability of coral occupancy

We qualitatively reviewed the mean proportion of corals occupied on each transect to determine whether cyclone activity would change the relative proportion of either social organization’s occupancy. Since we expected coral size to change with cyclone activity, we assessed whether coral size (a potential aspect of habitat quality) was related to the type of goby species (PF or GF) that occupied it during each survey. This was examined by assessing the multinomial probability that corals would be inhabited by either GF species, PF species or neither. These data were modeled as a multinomial response with coral size and survey time as predictors. Prior to cyclone Ita (survey 1), these data were only collected at one site (Palfrey; Fig 1). For each of the remaining time points (surveys 2–4), data were collected from various sites around Lizard Island (Fig 1; S1 Data). Misclassification error is the proportion of false classifications predicted by the model and was used to assess model performance. The multinomial model was conducted in R using the nnet package [75].

## Results

### Categorization of social organization

Of the 13 *Gobiodon* spp. surveyed at Lizard Island, five species were categorized as “GF” species and eight species were classified as “PF” (see above for definitions) (Table 1).

### Group size

Prior to cyclone Ita (Feb 2014), GF species were observed with mean group sizes of 2.71 (± 0.17 SE) individuals per coral. The mean group size of GF species decreased to 2.13 (± 0.11 SE) following cyclone Ita (Aug 2014). Five months later (Jan 2015, 9 months after cyclone Ita) the mean group size of GF species appeared to show some sign of recovery, increasing to 2.58 ± 0.11 (SE). This trend of recovering group sizes was not evident 10 months after cyclone Nathan (Jan 2016), when mean group sizes for GF species was 2.27 ± 0.15 (SE), similar to those just four months after cyclone Ita. Meanwhile PF species had a mean group size of 1.88 (± 0.05 SE) individuals per coral at the beginning of the study (Feb 2014) and maintained their group sizes at a similar level through both cyclones. The mean group sizes of PF species was 1.81 (± 0.03 SE), 1.74 (± 0.04 SE) and 1.74 (± 0.04 SE) for the Aug 2014, Jan 2015 and Jan 2016 surveys respectively.

**Fig 3:**
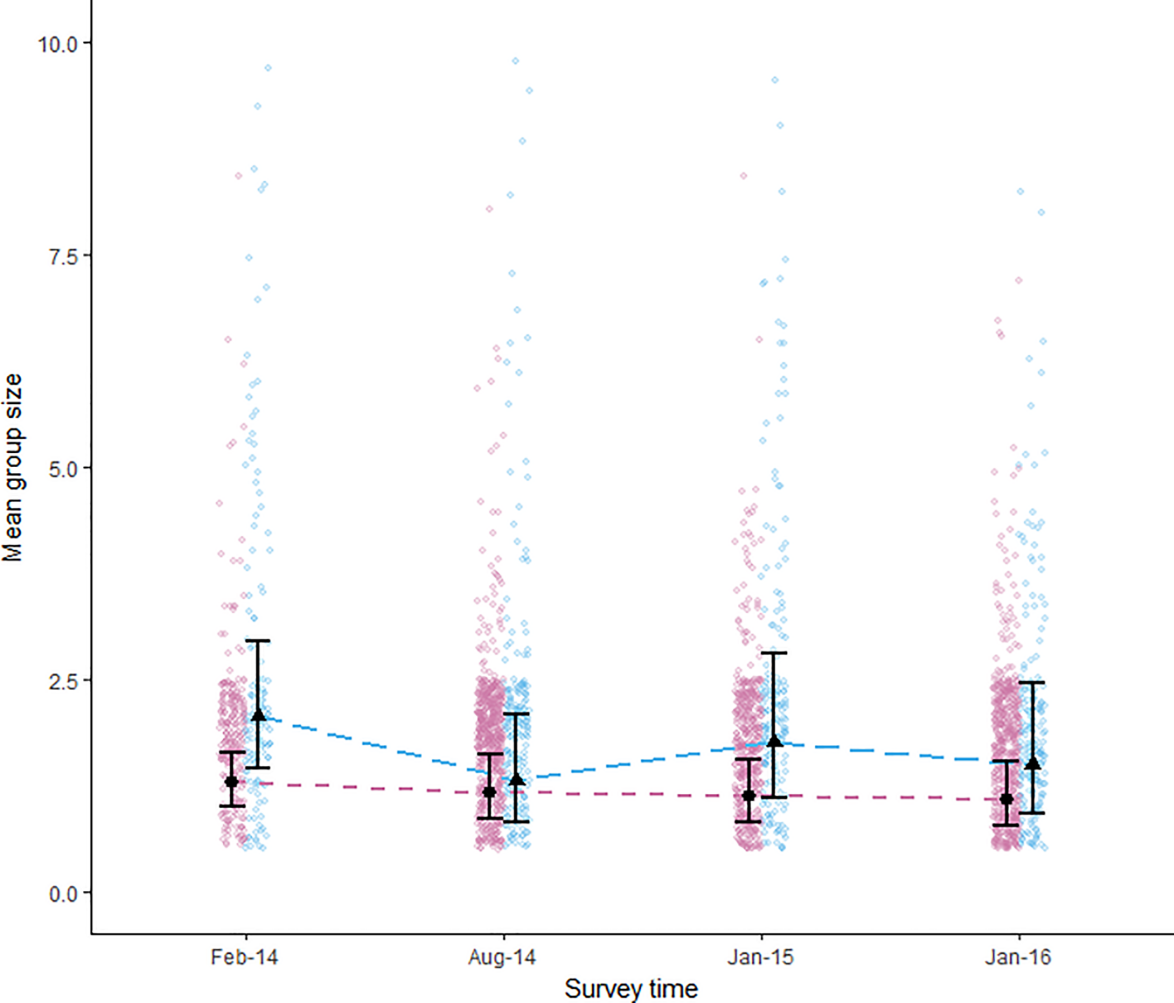
Variation in group size of PF and GF species in response to cyclone activity. Modeled mean group size of pair-forming (circles, pink dotted line) and group-forming (triangles, blue dashed line) species at the four survey times. Error bars are 95% CI. Cyclone symbols show when each cyclone impacted the research sites. Raw data for pair- (pink) and group-forming (blue) species are shown as jittered point clouds. Six observations of group sizes greater than 10 are not shown here, but were included in the model.

Group-forming species had larger mean group sizes than PF species at every survey time (Fig 3), although the difference in group size between GF and PF species reduced substantially following cyclone Ita (Aug 2014; Fig 3). This was due to the reduction in the mean group size of the GF species after cyclone Ita. These patterns were supported by the statistical model which had a RMSE of 1.36 (S1 Table). The model predicted an initial decrease in the mean group size of GF species following cyclone Ita (pairwise comparison ratio 1.58 (Feb-14/group:Aug-14/group, 95% CI (0.76, 1.83)). However, the predicted mean group sizes remained at these lower sizes for the subsequent surveys (S2 Table; Fig 3). The model did show a slight increase in mean group size of GF species in the Jan-15 survey (confidence interval was relatively large; estimated marginal mean 1.78, 95% CI (1.10, 2.81); Fig 3).

Mean group sizes of PF species did not change significantly throughout the study. The statistical model showed very little variation in group size during any survey time (Fig 3), but predicted lower mean group sizes than observed, ranging from 1.28 ± 0.16 (SE) before the cyclones to 1.09 ± 0.19 (SE) after both cyclones.

### Coral size

**Fig 4:**
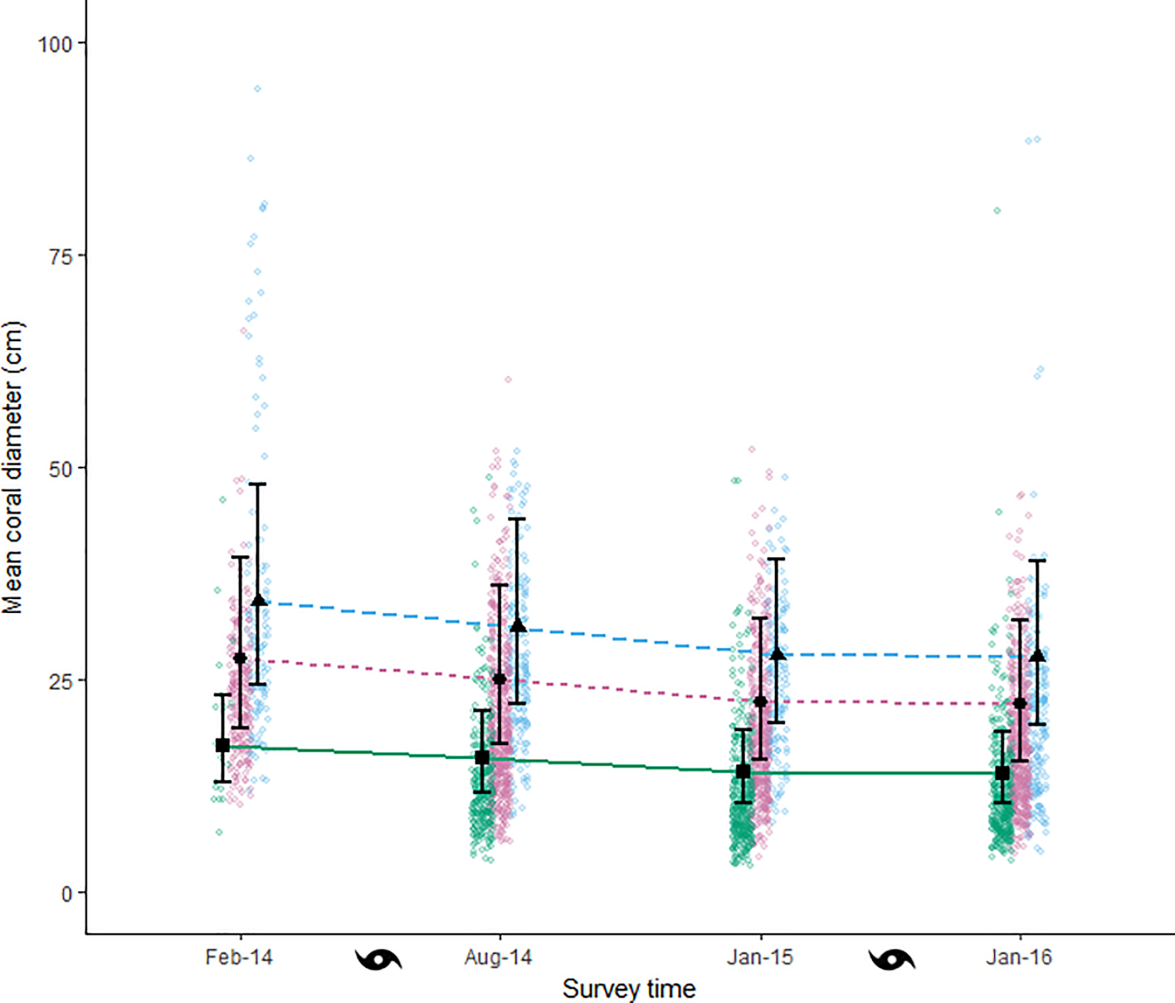
Mean coral size over the four survey times. Modeled mean coral diameter inhabited by pair-forming (circles, pink dotted line), group-forming (triangles, blue dashed line) species and vacant corals (squares, green solid line). Error bars are 95% CI. Cyclone symbols show when each cyclone impacted the research sites. Raw data of empty corals (green), pair- and group-forming species (pink and blue, respectively) are shown as jittered point clouds. Eight observations of corals larger than 100 cm mean diameter were omitted from this figure but were included in the model.

**Fig 5:**
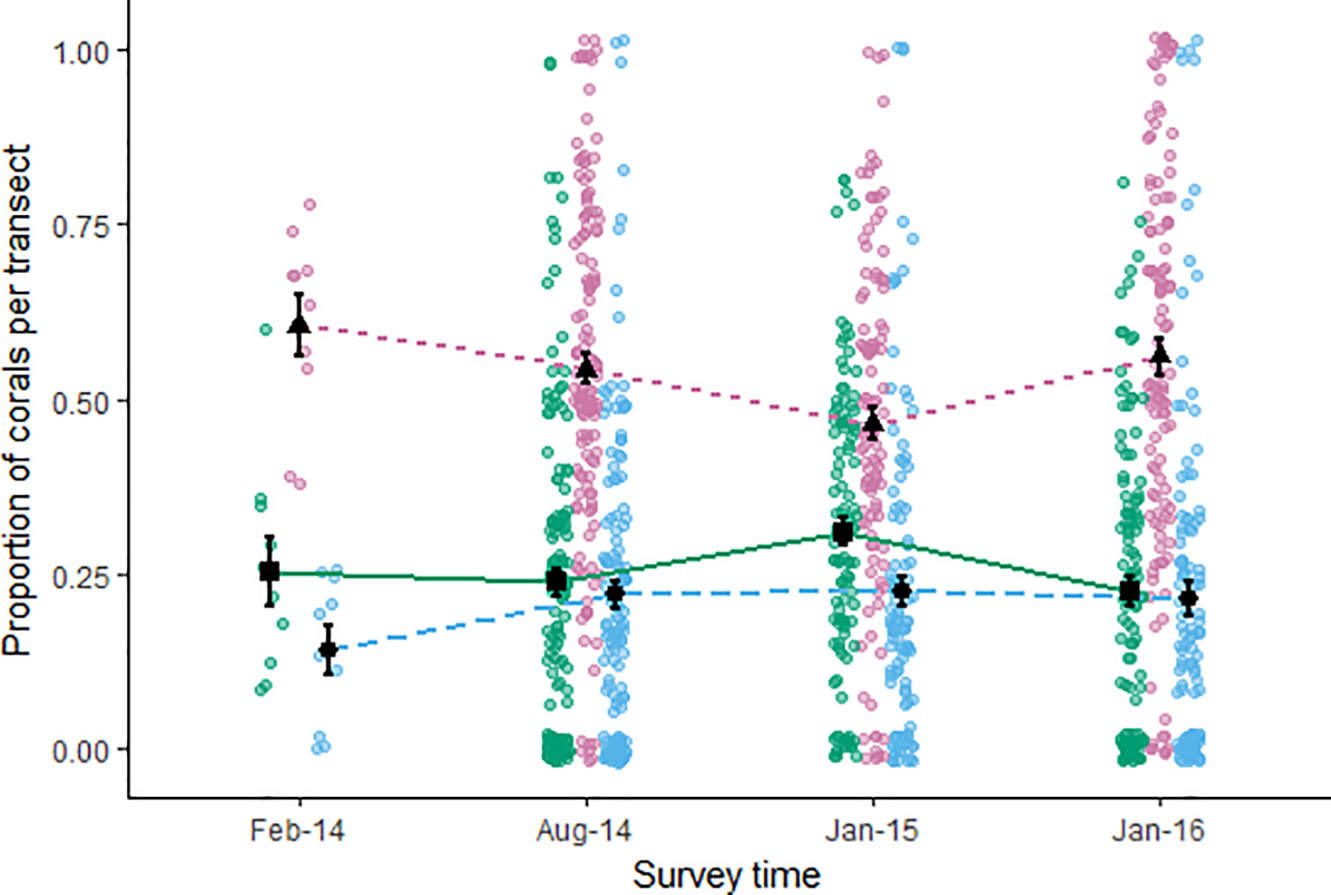
Mean proportion of corals occupied by each social organization and remaining vacant throughout the study. Mean proportion of corals inhabited by pair-forming species (triangles, pink dotted line), group-forming species (circles, blue dashed line) and remaining vacant (squares, green solid line) over the four surveys. Error bars indicate standard error. Raw data are shown as jittered point clouds for vacant (green), pair- (pink) and group-forming (blue) species.

Over each successive survey, the mean size of corals inhabited by GF species, PF species and the mean size of uninhabited corals all decreased (pairwise comparison ratio 1.23 (Feb-14:Jan16, 95% CI (1.16, 1.32); Fig 4). The number of very large corals (greater than 50 cm mean diameter) also decreased substantially following the first cyclone (cyclone Ita; Fig 4) and were detected in low numbers in all subsequent surveys. The interaction between sociality and survey time was not significant (analysis of deviance χ^2^ = 3.36, df = 3, P = 0.34), indicating that the coral size decreased at a similar rate across the four survey times for each category of social organization. As this interaction was non-significant, pairwise comparisons were conducted on the main effects only. On average, GF species inhabited larger corals (26.93 ± 0.56 (SE)) than the PF species (19.76 ± 0.19 (SE)) during each survey and the mean size of uninhabited corals (12.86 ± 0.25 (SE)) was always less than that of inhabited corals (Fig 4). The pattern of decreasing coral size was supported by the statistical model (RMSE = 7.42; Fig 4). The model also supported the pattern of GF species inhabiting larger corals than PF species on average (pairwise comparison ratio 0.80 (PF:GF), 95% CI (0.61, 1.06); Fig 5). Vacant corals were smaller than corals inhabited by either PF or GF species (pairwise comparison ratio 0.63 (vacant:PF), 95% CI (0.37, 0.85); pairwise comparison ratio 0.51 (vacant:GF), 95% CI (0.37, 0.69) respectively).

To assess whether habitat saturation (an ecological constraint) was acting as a constraint on subordinate dispersal, we looked at whether the number of vacant corals differed between transects with or without groups (colonies with 3 or more individuals) of GF species. Corals that were uninhabited were present on transects where at least one group of GF species was present (S1 Data). This means that there was vacant habitat available for subordinates to disperse to. However, there was no difference in the mean number of empty corals on transects with or without a group of GF species during any survey time detected by the model (pairwise comparison ratio 1.19 (no groups:groups present), 95% CI (0.89, 1.44). This could indicate that some coral vacancy was due to reduced abundance of coral gobies overall, but the fact that groups of GF species were present on transects where there were corals available to disperse to demonstrates that either; some constraint was restricting dispersal from the group or subordinate gobies were receiving a benefit from remaining within the group.

### Proportion of inhabited corals and probability of occupation

PF species occupied proportionally more corals on average during each survey than GF species (Fig 5). There was a similar proportion of corals occupied by GF species as there were vacant corals during each survey. The proportion of corals inhabited by PF species decreased from 0.61 ± 0.04 (SE) at the beginning of the study (Feb 2014) to 0.54 ± 0.02 (SE) after cyclone Ita (Aug 2014). This downward trend continued into the next survey (Jan 2015) where the proportion of corals inhabited by PF species was 0.46 ± 0.02 (SE). However, the proportion of corals inhabited by PF species increased after cyclone Nathan (Jan 2016) to 0.56 ± 0.03 (SE). The GF species on the other hand showed relative stability in the proportion of corals they occupied during the study. There was an initial increase in the proportion of corals inhabited by GF species from 0.14 ± 0.03 (SE) at the beginning of the study to 0.22 ± 0.02 (SE) after cyclone Ita (Aug 2014). The mean proportion of corals occupied by GF species then remained at similar levels for the remaining two surveys (Fig 5). The proportion of vacant corals was also relatively unchanged throughout the study except for a small increase nine months after cyclone Ita (Jan 2015; 0.31 ± 0.02 (SE); Fig 5).

**Fig 6:**
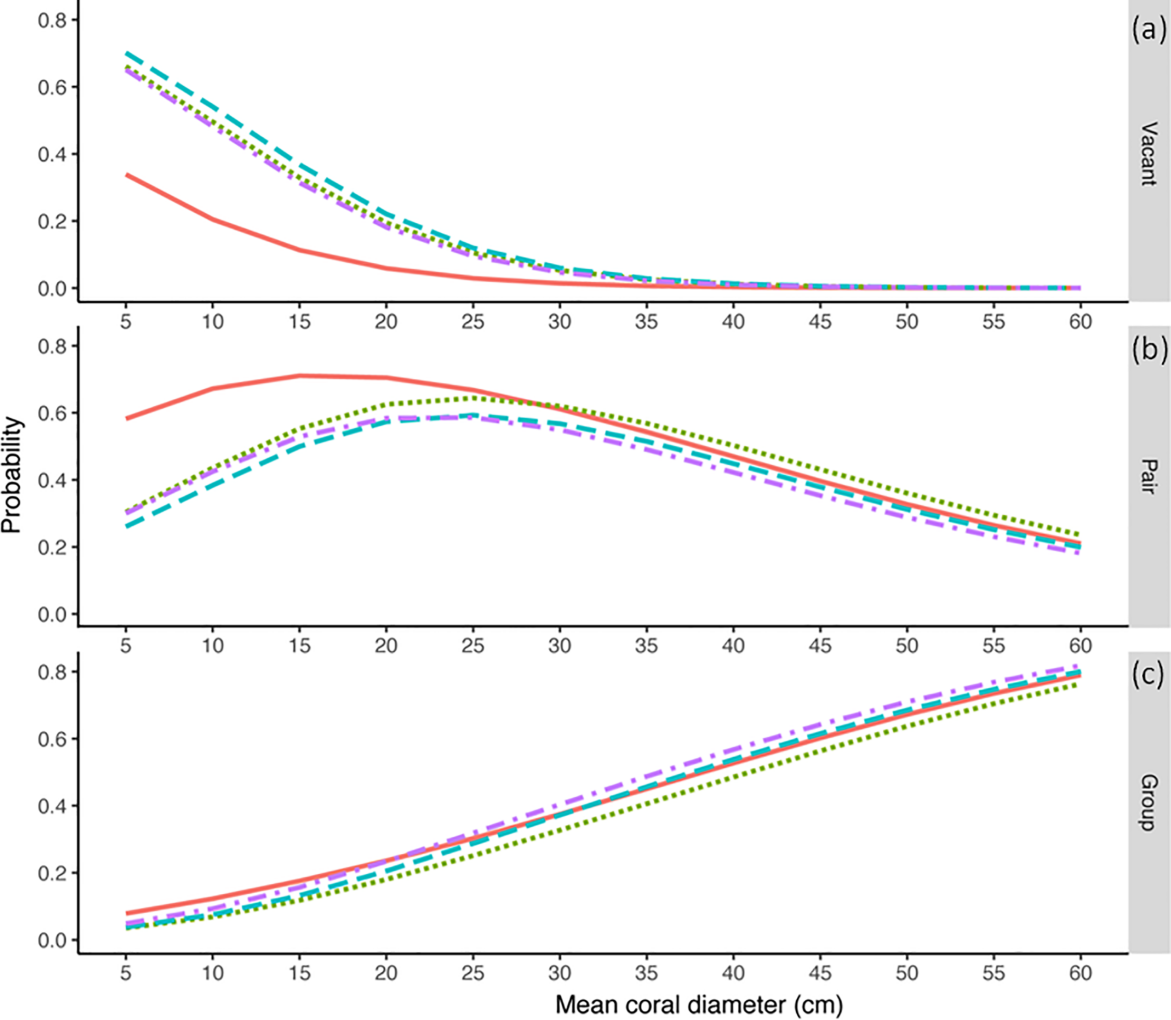
Probability of occupation for corals of varying mean diameter. Probability that a coral of given size would remain vacant (a) or be inhabited by either a pair- (b) or group-forming (c) species of *Gobiodon*. Probabilities are shown for each survey time: Feb 2014 (orange, unbroken), Aug 2014 (green, dotted), Jan 2015 (blue, dashed), Jan 2016 (purple, dot-dash).

The multinomial model of coral occupancy had a misclassification rate of 0.404 indicating that the predictions may not be reliable. Nevertheless, the trends agree reasonably well with our observations and we give a qualitative account of these, recognizing that probability estimates may have large error. Odds ratios and associated confidence intervals for the model coefficients are available in S3 Table, however, we urge the same caution in their interpretation. Prior to cyclone activity (February 2014), there was a low probability that the smallest corals would remain vacant and this probability decreased rapidly for corals of increasing mean diameter (Fig 6A). This was consistent with our observations as larger corals were rarely vacant (Fig 4, pink and blue points). After cyclone Ita, there was a similar pattern of decreasing probability of corals remaining vacant with increasing coral size (Fig 6A), but there was a higher probability of the smallest corals remaining vacant. Again, this pattern was consistent with our observations of coral size (Fig 4). The probability that a PF species would occupy a coral increased initially with increasing coral size, but then decreased after reaching an apparent optimal coral size around 15 cm (Fig 6B, solid orange line). This pattern of increasing to an optimum size is certainly plausible if we consider that GF species typically inhabited the larger corals (Fig 4) posing an upper restraint on occupancy by PF species. Corals in the smaller range may have been less desirable as they may not support successful feeding, reproduction or protection from predators. Furthermore, the coral size model had predicted the mean coral size for PF species within this coral size range (Fig 4). The ‘optimal’ coral size for PF species appeared to increase to 20 cm– 30 cm in the survey times after cyclone Ita (Fig 6B). Consistent with the concept of the PF species having lower probability of occupancy at higher coral sizes, the probability that a GF species would occupy a coral increased as coral size increased (Fig 6C). This relationship between coral size and probability of inhabitance by a GF species did not change with respect to survey time (Fig 6C).

## Discussion

The effects of cyclones on the social organization of coral-reef fish are poorly understood despite clear links between social organization and factors that could affect species persistence and recovery following environmental disturbances [1–3]. Here, we investigated the impacts of two successive cyclones (Ita 2014 and Nathan 2015) on the social organization of coral-gobies over three years, and at the same time shed light on the possible factors influencing the formation of social groups.

### Effects of cyclones on social organization and coral size

Both cyclones had a small, but detectable effect on the social organization of GF species. Similar impacts on social organization were not evident in the PF species. The group size of GF species declined, while the group sizes of PF species showed little variation over time. Despite the general decline in their group sizes, GF species exhibited some recovery eight months after cyclone Ita. However, there was no such recovery exhibited after cyclone Nathan. The lack of apparent recovery after cyclone Nathan indicates that multiple impacts of this nature can have longer lasting negative impacts on the social structure of GF species. The relative stability of group sizes in the PF species on the other hand, suggests a level of resilience in social structure in the face of natural disturbance. Overall, mean coral size and the presence of very large corals (greater than 50 cm mean diameter) decreased with each cyclone. This was consistent with damage reported in studies on these cyclones [60–63] and others [45].

### Implications for pair- and group-forming species

The overall reduction in coral sizes meant that both GF and PF species were more frequently observed in corals of smaller sizes including some of a size that were unoccupied before the cyclones (Feb 2014). Therefore, the recovery in group size of GF species following cyclone Ita (Jan 2015) occurred despite the fact that the corals they inhabited were smaller on average compared to pre-cyclone (Feb 2014). This result was unexpected, given the positive relationship between coral size and group size regularly reported for social habitat-specialist reef fishes [55, 77, 78]. This may indicate that GF species of gobies will tolerate greater coral saturation (i.e. more subordinates in smaller corals) following a disturbance, especially if they benefit in future reproduction or survival from doing so [79].

Despite this small recovery following cyclone Ita, group sizes of GF species remained relatively lower following cyclone Nathan. This may be due to social conflict [6] and recruitment prevention [37], demonstrated in other social fishes at high rates of habitat saturation. Smaller group sizes suggest lower numbers of subordinates which may have a negative impact on future reproductive efforts [79]. Smaller group sizes could also be problematic under a regime of repeated disturbance as larger group size may provide a level of redundancy and buffer effects of future disturbance [26, 42, 43]. However, when group sizes are reduced, so too is this redundancy.

The proportion of corals inhabited by PF species did decrease following cyclone Ita, but had returned to pre-cyclone levels in the period following cyclone Nathan. At all survey times, PF species inhabited a substantially higher proportion of corals than GF species. This suggests that PF species might be better able to colonize vacant corals than GF species, for example by out-competing GF species for habitat [57, 80]. However, most of the GF species in our study tended to prefer different species of coral to the PF species and we therefore consider competitive effects unlikely. Instead, the greater proportion of corals inhabited by PF species could be due to their tendency to live in intermediate sized (20–30 cm) corals as shown by our analysis of the probability of occupation by a PF species. Corals in this size range were relatively common in the surveys following cyclone Ita (compared to the larger corals that GF species tend to inhabit). Group-forming species on the other hand showed a relatively lower probability of occupying corals in this intermediate size range. This ability or preference of PF species to occupy corals in the range of sizes most commonly found after the cyclones could be advantageous at the population scale, as long as these habitats were of sufficient quality to enable foraging, protection from predators and successful breeding [58, 59].

### Ecological constraints and benefits of philoparty

In theory, subordinates living in a group could maximize their lifetime reproductive success if they dispersed to pursue independent breeding rather than remaining in a group as a subordinate. In practice however, various ecological constraints and benefits of remaining philopatric amongst other factors (e.g. life-history and phylogeny), alter the advantages of dispersing from or remaining in their current group [11, 12, 20]. For example, a lack of vacant habitat to disperse to in order to pursue independent breeding (an ecological constraint) would increase the benefit of remaining in the group, even as a non-breeding subordinate, especially if the subordinate stands to inherit the breeding position in the future (a benefit of philopatry). Habitat saturation (i.e. lack of available suitable habitat) is often invoked as a key ecological constraint leading to group formation and maintenance in a variety of taxa (e.g. birds [13]; mammals [81]; fish [20]). Other studies on a closely related coral goby [23] and on social freshwater fishes [22] have found the combination of habitat saturation and benefits of philopatry promote group-living. However, we found little evidence to support habitat saturation acting as a constraint on dispersal in coral gobies following these disturbances.

Our analysis of vacant corals on transects with and without groups of GF species indicate that GF groups were present even when alternative corals were available for subordinate dispersal. As we only included corals of a size that pairs of gobies had been observed in, these alternative corals are assumed to be of a size capable of supporting at least a breeding pair. Our study therefore indicates that habitat saturation alone was unlikely to explain group formation. Instead, and consistent with the benefits of philopatry hypothesis, subordinates of GF species stayed within the group, presumably obtaining benefits that group living provides (e.g. inheritance of a breeding position in a good quality habitat).

Additionally, our analysis of the probability of occupation showed that GF species were increasingly more likely to inhabit a coral as coral size increased. Coral size has been shown to be related to individual growth, survival and reproductive success in some coral-associated fishes and may therefore be considered a reasonable proxy for habitat quality [58, 59]. This strong association between coral size (quality) and probability of occupancy by a GF species is consistent with the benefits of philopatry model as we would expect larger group sizes (characteristic of more social species) in higher-quality habitat. Conversely, under a habitat saturation model we would expect a much weaker association between coral size and the probability of occupancy by a GF species as subordinates would be expected to disperse to vacant habitat of any size that could support independent breeding.

Furthermore, if habitat saturation (availability of corals) was acting as a constraint on dispersal following the cyclone, we would expect the proportion of inhabited corals to approach 100% as subordinates would quickly fill any vacant habitat to pursue independent breeding [13]. Alternatively, if there were sufficient benefits of residing in a high-quality habitat, we would expect the proportion of inhabited corals to be substantially lower than 100% after the cyclones as individuals living in low-quality habitat would vacate and take up residence in a higher-quality habitat as a subordinate. We found the proportion of corals inhabited by social species was very low and relatively constant (< 25%) throughout the study, even though there were vacant corals present (approximately 20% per transect), suggesting that benefits of philopatry and not habitat saturation was responsible for group formation.

## Conclusion

Few studies thus far have examined the effects that extreme climatic events such as tropical cyclones could have on social organization of social species. While two cyclones in consecutive years may be rare, the frequency of the most intense cyclones is projected to increase as sea surface temperatures continue to rise in the future and repeated disturbances may become more prevalent [82]. The destructive nature of these events on coral-reef communities has been well documented [46, 48, 83]. However, changes to social organization from such events have been less studied. Here we demonstrated that repeated cyclones are likely to negatively impact social organization in a genus of coral-reef fishes through flow-on effects of the destruction of habitat, but only in GF species. Pair-forming species appear to be able to monopolize smaller corals and maintain their social organization in response to extreme climatic events. Additionally, we suggest that the most likely mechanism for the maintenance of group sizes in GF species are benefits of philopatry, but these benefits only promote group living when the habitat is of sufficient size. Cyclones are capable of reducing whole areas of coral to well below what appears to be the minimum size threshold for GF coral gobies to form their usual group structures, which may be linked to their ability to recover from such disasters. In fact, we observed several sites that were completely devoid of corals (and hence coral gobies) following each cyclone. With the frequency of more intense cyclones and other stressors on coral reefs (e.g. coral bleaching) set to increase in the near future, population declines and localized extinctions of GF species of coral gobies through habitat loss and lowered recovery ability due to impacts on their social organization are a real possibility. While PF species appear to buffer these effects somewhat, they are still vulnerable to habitat destruction caused by these catastrophic events.

## Supporting information

S1 Data Raw Data. Data collected from Lizard Island over four survey times between February 2014 and February 2016. Includes some data from collected by MW and SK in 2013. This 2013 data was only used in the categorization of *Gobiodon* species, not in the statistical models.
(CSV)

S1 Fig Sociality index for each species of *Gobiodon* observed at Lizard Island. Sociality indices (red dot) calculated for each species. Jittered point clouds indicate the relative number of colonies that were available to calculate the index from. There is a natural split in the species’ indices around 0.5.
(DOCX)

S2 Fig *Gobiodon* juvenile abundance at Lizard Island. Predicted mean juvenile abundance and 95% CI for pair- and group-forming species (pink and blue respectively) across each survey time. Raw data is shown as jittered point clouds.
(DOCX)

S1 Table Summary of statistical models. Results from statistical models. Abbreviations are: Akaike Information Criterion (AIC); standard error (SE); degrees of freedom (df); standard deviation (SD); root mean squared error (RMSE).
(DOCX)

S2 Table Pairwise comparisons for the fixed effects terms of each of the group size, coral size, empty corals and predicted probabilities of inhabitance. Pairwise comparisons were conducted in R using the emmeans package [1]. For a given contrast A/B, ratios greater than 1.00 indicate that A is greater than B and ratios less than 1.00 indicate that B is greater than A.
(DOCX)

S3 Table Model coefficients for the multinomial probability of occupancy. Odds and associated confidence interval (CI) for each model coefficient. Vacant corals were the reference group. Odds = 1 indicate an equal chance that the coral would remain vacant or be inhabited by either a pair- or group-forming species. Odds > 1 indicate a greater chance of the coral being inhabited by either pair- or group-forming species rather than remaining vacant. Odds < 1 indicates a greater chance of the coral remaining vacant.
(DOCX)
