# Supplementary material for: Repeated cyclone events reveal potential causes of sociality in coral-dwelling Gobiodon fishes
Source: PLoS One. 2018 Sep 5;13(9):e0202407. doi: 10.1371/journal.pone.0202407 (PMC6124712; doi:10.1371/journal.pone.0202407)
Supplement: S1 Fig — Sociality indices (red dot) calculated for each species. Jittered point clouds indicate the relative number of colonies that were available to calculate the index from. There is a natural split in the species’ indices around 0.5. (DOCX) [file pone.0202407.s002.docx]

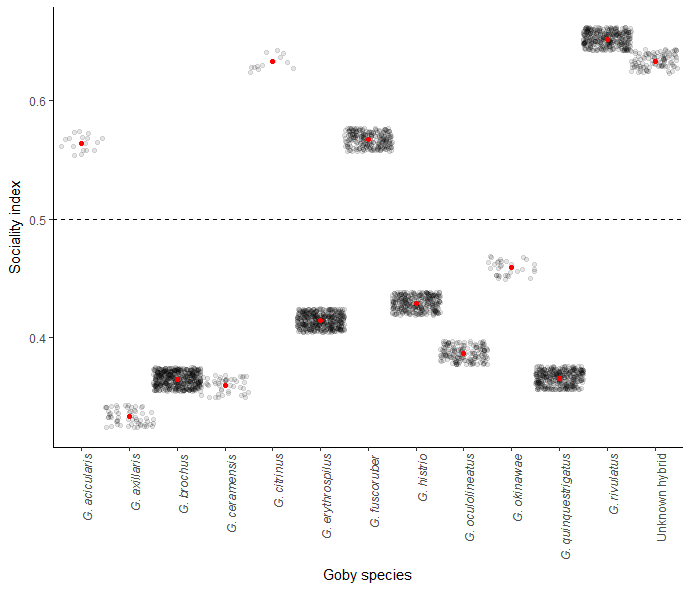


**S1 Fig: Sociality index for each species of *Gobiodon* observed at Lizard Island.** Sociality indices (red dot) calculated for each species. Jittered point clouds indicate the relative number of colonies that were available to calculate the index from. There is a natural split in the species’ indices around 0.5.
