# Supplementary material for: Repeated cyclone events reveal potential causes of sociality in coral-dwelling Gobiodon fishes
Source: PLoS One. 2018 Sep 5;13(9):e0202407. doi: 10.1371/journal.pone.0202407 (PMC6124712; doi:10.1371/journal.pone.0202407)
Supplement: S2 Fig — Predicted mean juvenile abundance and 95% CI for pair- and group-forming species (pink and blue respectively) across each survey time. Raw data is shown as jittered point clouds. (DOCX) [file pone.0202407.s003.docx]

**S2. Number of Juveniles in the Lizard Island population of *Gobiodon***

We recorded juvenile number when they were present in coral colonies. However they were excluded from the group size analyses because identification of many species’ juveniles is not possible from morphological features and we observed several moving between multiple corals. This meant that we could not definitively assign them to any particular group. We assessed whether juvenile abundance changed over successive survey times for pair- and group-forming species (identification assumed qualitatively from the colony they appeared to spend the most time with) with a generalized linear mixed model. The model contained juvenile abundance as the response variable, survey time and social category as predictors and site, goby species and coral species as random effects. We used a zero inflated negative binomial model as the data set was heavily zero-inflated (S4 Data) and the negative binomial model produced the best fitting model when compared with a zero-inflated Poisson model (negative binomial AIC = 2163.74, Poisson AIC = 2197.58). The model fit was assessed using root mean square error (RMSE) which is a measure of the overall error between the model predictions and the raw data in the units of the response variable. The model fit was reasonable given the true range of the samples but produced very large confidence intervals for group-forming species in the last two survey times (RMSE = 1.06; Fig 1). Nevertheless, we are confident that the abundance of juveniles for both pair- and group-forming species was similar at all survey times and that juvenile recruitment played a very minor role in the group size patterns we reported.


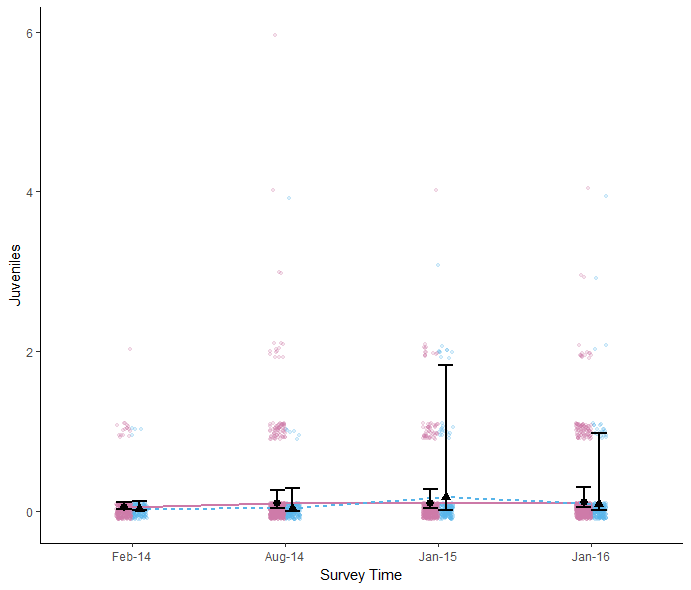


**S2 Fig: *Gobiodon* juvenile abundance at Lizard Island.** Predicted mean juvenile abundance and 95% CI for pair- and group-forming species (pink and blue respectively) across each survey time. Raw data is shown as jittered point clouds.
