# Supplementary material for: Repeated cyclone events reveal potential causes of sociality in coral-dwelling Gobiodon fishes
Source: PLoS One. 2018 Sep 5;13(9):e0202407. doi: 10.1371/journal.pone.0202407 (PMC6124712; doi:10.1371/journal.pone.0202407)
Supplement: S1 Table — Results from statistical models. Abbreviations are: Akaike Information Criterion (AIC); standard error (SE); degrees of freedom (df); standard deviation (SD); root mean squared error (RMSE). (DOCX) [file pone.0202407.s004.docx]

**S1. Table: Summary of statistical models**

| **Model** | **AIC** | **Performance** | **Response** | **Distribution** | **Parameter** | | **Estimate** | **SE** | **Fixed Effects** | **Coefficient** | **Estimate** | **SE** | **df** | **Random Effects** | **SD** |
| --- | --- | --- | --- | --- | --- | --- | --- | --- | --- | --- | --- | --- | --- | --- | --- |
| Group size (interaction) | 7867.8 | 0.843 (RMSE) | Group Size (count) | Negative binomial | Dispersion | | 403.430 | 0.272 | Survey | Intercept (Feb-14) | 0.664 | 0.063 | 2574 | Site | 0.015 |
|  |  |  |  |  |  |  |  |  |  | Aug-14 | -0.041 | 0.058 | 2574 | Goby spp | 2E-04 |
|  |  |  |  |  |  |  |  |  |  | Jan-15 | -0.067 | 0.060 | 2574 | Coral spp | 0.137 |
|  |  |  |  |  |  |  |  |  |  | Jan-16 | -0.073 | 0.058 | 2574 |  |  |
|  |  |  |  |  |  |  |  |  | Sociality | Group | 0.290 | 0.075 | 2574 |  |  |
|  |  |  |  |  |  |  |  |  | Interact | Aug-14:Group | -0.202 | 0.092 | 2574 |  |  |
|  |  |  |  |  |  |  |  |  |  | Jan-15:Group | 0.027 | 0.093 | 2574 |  |  |
|  |  |  |  |  |  |  |  |  |  | Jan-16:Group | -0.084 | 0.093 | 2574 |  |  |
| Group size (interaction) | 6307.2 | 1.160  (RMSE) | Group Size (count) | Zero-truncated negative binomial | Dispersion | | 8.049 | 1.599 | Survey | Intercept (Feb-14) | 0.251 | 0.127 | 2574 | Site | 0.157 |
|  |  |  |  |  |  |  |  |  |  | Aug-14 | -0.090 | 0.089 | 2574 | Goby spp | 0.169 |
|  |  |  |  |  |  |  |  |  |  | Jan-15 | -0.136 | 0.094 | 2574 | Coral spp | 0.268 |
|  |  |  |  |  |  |  |  |  |  | Jan-16 | -0.160 | 0.092 | 2574 |  |  |
|  |  |  |  |  |  |  |  |  | Sociality | Group | 0.476 | 0.151 | 2574 |  |  |
|  |  |  |  |  |  |  |  |  | Interact | Aug-14:Group | -0.367 | 0.135 | 2574 |  |  |
|  |  |  |  |  |  |  |  |  |  | Jan-15:Group | -0.025 | 0.138 | 2574 |  |  |
|  |  |  |  |  |  |  |  |  |  | Jan-16:Group | -0.162 | 0.137 | 2574 |  |  |
| Coral Size (interaction) | 23321 | 7.387 (RMSE) | Mean coral diameter (continuous) | Gamma | Shape | | 7.654 | 0.179 | Sociality | Intercept (Vacant) | 2.927 | 0.169 | 3537 | Site | 0.160 |
|  |  |  |  |  |  |  |  |  |  | Pair | 0.385 | 0.153 | 3537 | Goby spp | 0.116 |
|  |  |  |  |  |  |  |  |  |  | Group | 0.594 | 0.156 | 3537 | Coral spp | 0.383 |
|  |  |  |  |  |  |  |  |  | Survey | Aug-14 | -0.158 | 0.087 | 3537 |  |  |
|  |  |  |  |  |  |  |  |  |  | Jan-15 | -0.356 | 0.087 | 3537 |  |  |
|  |  |  |  |  |  |  |  |  |  | Jan-16 | -0.227 | 0.088 | 3537 |  |  |
|  |  |  |  |  |  |  |  |  | Interact | Pair:Aug-14 | 0.061 | 0.093 | 3537 |  |  |
|  |  |  |  |  |  |  |  |  |  | Group:Aug-14 | 0.087 | 0.098 | 3537 |  |  |
|  |  |  |  |  |  |  |  |  |  | Pair:Jan-15 | 0.178 | 0.093 | 3537 |  |  |
|  |  |  |  |  |  |  |  |  |  | Group:Jan-15 | 0.218 | 0.098 | 3537 |  |  |
|  |  |  |  |  |  |  |  |  |  | Pair:Jan-16 | 0.000 | 0.093 | 3537 |  |  |
|  |  |  |  |  |  |  |  |  |  | Group:Jan-16 | -0.028 | 0.099 | 3537 |  |  |
|  |  |  |  |  |  | |  |  |  |  |  |  |  |  |  |
|  |  |  |  |  |  | |  |  |  |  |  |  |  |  |  |
|  |  |  |  |  |  | |  |  |  |  |  |  |  |  |  |
|  |  |  |  |  |  | |  |  |  |  |  |  |  |  |  |
| Coral Size (main effects) | 23343 | 7.423 (RMSE) | Mean coral diameter (continuous) | Gamma | Shape | | 7.585 | 0.178 | Sociality | Intercept (Vacant) | 2.850 | 0.150 | 3543 | Site | 0.163 |
|  |  |  |  |  |  |  |  |  |  | Pair | 0.463 | 0.128 | 3543 | Goby spp | 0.380 |
|  |  |  |  |  |  |  |  |  |  | Group | 0.683 | 0.132 | 3543 | Coral spp | 0.118 |
|  |  |  |  |  |  |  |  |  | Survey | Aug-14 | -0.091 | 0.025 | 3543 |  |  |
|  |  |  |  |  |  |  |  |  |  | Jan-15 | -0.206 | 0.026 | 3543 |  |  |
|  |  |  |  |  |  |  |  |  |  | Jan-16 | -0.213 | 0.025 | 3543 |  |  |
| Transects with/without groups (interaction) | 1513.1 | 1.440 (RMSE) | vacant corals (count) | Zero-inflated Poisson | Zero-inflation | | 0.2261 | 0.027 | Survey | Intercept (Feb-14) | 0.588 | 0.333 | 366 |  |  |
|  |  |  |  |  |  |  |  |  |  | Aug-14 | 0.288 | 0.343 | 366 |  |  |
|  |  |  |  |  |  |  |  |  |  | Jan-15 | 0.617 | 0.342 | 366 |  |  |
|  |  |  |  |  |  |  |  |  |  | Jan-16 | 0.146 | 0.347 | 366 |  |  |
|  |  |  |  |  |  |  |  |  | w/w.out groups | w/groups | 0.105 | 0.459 | 366 |  |  |
|  |  |  |  |  |  |  |  |  | Interact | Aug-14: w/groups | -0.239 | 0.492 | 366 |  |  |
|  |  |  |  |  |  |  |  |  |  | Jan-15: w/groups | -0.395 | 0.482 | 366 |  |  |
|  |  |  |  |  |  |  |  |  |  | Jan-16: w/groups | 0.267 | 0.495 | 366 |  |  |
|  |  |  |  |  |  |  | |  |  |  |  |  |  |  |  |
|  |  |  |  |  |  |  | |  |  |  |  |  |  |  |  |
| Transects with/without groups (interaction) | 1422 | 1.187 (RMSE) | vacant corals (count) | Zero-inflated negative binomial | Zero-inflation | 1.061E-06 | | 0.000 | Survey | Intercept (Feb-14) | 0.830 | 0.405 | 365 |  |  |
|  |  |  |  |  |  |  |  |  |  | Aug-14 | -0.244 | 0.416 | 365 |  |  |
|  |  |  |  |  | Dispersion | 2.429 | | 0.216 |  | Jan-15 | 0.151 | 0.418 | 365 |  |  |
|  |  |  |  |  |  |  | |  |  | Jan-16 | -0.319 | 0.420 | 365 |  |  |
|  |  |  |  |  |  |  | |  | w/w.out groups | w/groups | 0.083 | 0.561 | 365 |  |  |
|  |  |  |  |  |  |  | |  | Interact | Aug-14: w/groups | -0.127 | 0.605 | 365 |  |  |
|  |  |  |  |  |  |  | |  |  | Jan-15: w/groups | -0.342 | 0.594 | 365 |  |  |
|  |  |  |  |  |  |  | |  |  | Jan-16: w/groups | -0.100 | 0.612 | 365 |  |  |
|  |  |  |  |  |  | |  |  |  |  |  |  |  |  |  |
| Probability of inhabitance (main effects) | 6277.7 | 0.492 (misclass-ification) | Coral occupant | Multinomial | NONE | | NA | NA | Survey | Pair (Intercept) | -0.107 | 0.278 | NA |  |  |
|  |  |  |  |  |  | |  |  |  | Pair(Aug-14) | -1.318 | 0.256 | NA |  |  |
|  |  |  |  |  |  | |  |  |  | Pair(Jan-15) | -1.531 | 0.256 | NA |  |  |
|  |  |  |  |  |  | |  |  |  | Pair(Jan-16) | -1.315 | 0.256 | NA |  |  |
|  |  |  |  |  |  | |  |  | mean coral diam | Pair (Avg.Diam) | 0.130 | 0.007 | NA |  |  |
|  |  |  |  |  |  | |  |  | Survey | Group (Intercept) | -2.409 | 0.307 | NA |  |  |
|  |  |  |  |  |  | |  |  |  | Group (Aug-14) | -1.469 | 0.276 | NA |  |  |
|  |  |  |  |  |  | |  |  |  | Group (Jan-15) | -1.464 | 0.277 | NA |  |  |
|  |  |  |  |  |  | |  |  |  | Group (Jan-16) | -1.134 | 0.276 | NA |  |  |
|  |  |  |  |  |  | |  |  | mean coral diam | Group (Avg.Diam) | 0.190 | 0.008 | NA |  |  |

Results from statistical models. Abbreviations are: Akaike Information Criterion (AIC); standard error (SE); degrees of freedom (df); standard deviation (SD); root mean squared error (RMSE).
