# Supplementary material for: Repeated cyclone events reveal potential causes of sociality in coral-dwelling Gobiodon fishes
Source: PLoS One. 2018 Sep 5;13(9):e0202407. doi: 10.1371/journal.pone.0202407 (PMC6124712; doi:10.1371/journal.pone.0202407)
Supplement: S2 Table — Pairwise comparisons were conducted in R using the emmeans package [1]. For a given contrast A/B, ratios greater than 1.00 indicate that A is greater than B and ratios less than 1.00 indicate that B is greater than A. (DOCX) [file pone.0202407.s005.docx]

**S2 Table. Pairwise comparisons for the fixed effects terms of each of the group size, coral size, empty corals and predicted probabilities of inhabitance.**

Pairwise comparisons were conducted in R using the emmeans package [1]. For a given contrast A/B, ratios greater than 1.00 indicate that A is greater than B and ratios less than 1.00 indicate that B is greater than A.

**Table 1. Pairwise comparisons of interacting effects of sociality and survey time on group size.**

| **Contrast** | **Ratio** | **Lower CI** | **Upper CI** |
| --- | --- | --- | --- |
| Feb-14,AS / Aug-14,AS | 1.094 | 0.835 | 1.434 |
| Feb-14,AS / Jan-15,AS | 1.146 | 0.861 | 1.526 |
| Feb-14,AS / Jan-16,AS | 1.174 | 0.887 | 1.553 |
| Feb-14,AS / Feb-14,S | 0.622 | 0.394 | 0.981 |
| Feb-14,AS / Aug-14,S | 0.982 | 0.521 | 1.850 |
| Feb-14,AS / Jan-15,S | 0.730 | 0.388 | 1.375 |
| Feb-14,AS / Jan-16,S | 0.858 | 0.445 | 1.654 |
| Aug-14,AS / Jan-15,AS | 1.047 | 0.715 | 1.535 |
| Aug-14,AS / Jan-16,AS | 1.072 | 0.728 | 1.580 |
| Aug-14,AS / Feb-14,S | 0.568 | 0.329 | 0.981 |
| Aug-14,AS / Aug-14,S | 0.897 | 0.485 | 1.659 |
| Aug-14,AS / Jan-15,S | 0.667 | 0.334 | 1.333 |
| Aug-14,AS / Jan-16,S | 0.784 | 0.382 | 1.610 |
| Jan-15,AS / Jan-16,AS | 1.024 | 0.687 | 1.525 |
| Jan-15,AS / Feb-14,S | 0.542 | 0.313 | 0.940 |
| Jan-15,AS / Aug-14,S | 0.856 | 0.428 | 1.714 |
| Jan-15,AS / Jan-15,S | 0.637 | 0.340 | 1.193 |
| Jan-15,AS / Jan-16,S | 0.748 | 0.367 | 1.528 |
| Jan-16,AS / Feb-14,S | 0.530 | 0.303 | 0.925 |
| Jan-16,AS / Aug-14,S | 0.836 | 0.412 | 1.699 |
| Jan-16,AS / Jan-15,S | 0.622 | 0.308 | 1.257 |
| Jan-16,AS / Jan-16,S | 0.731 | 0.384 | 1.393 |
| Feb-14,S / Aug-14,S | 1.579 | 0.999 | 2.496 |
| Feb-14,S / Jan-15,S | 1.175 | 0.755 | 1.829 |
| Feb-14,S / Jan-16,S | 1.380 | 0.869 | 2.193 |
| Aug-14,S / Jan-15,S | 0.744 | 0.403 | 1.373 |
| Aug-14,S / Jan-16,S | 0.874 | 0.456 | 1.676 |
| Jan-15,S / Jan-16,S | 1.175 | 0.632 | 2.182 |

Pairwise comparisons conducted in R using the emmeans package. Tests were conducted on the log scale. Confidence intervals were back-transformed from the log scale.

**Table 2. Pairwise comparisons of main effects of sociality and survey time on coral size.**

| **Contrast** | **Ratio** | **Lower CI** | **Upper CI** |
| --- | --- | --- | --- |
| Feb-14 / Aug-14 | 1.096 | 1.028 | 1.168 |
| Feb-14 / Jan-15 | 1.228 | 1.150 | 1.312 |
| Feb-14 / Jan-16 | 1.237 | 1.160 | 1.319 |
| Aug-14 / Jan-15 | 1.121 | 1.026 | 1.225 |
| Aug-14 / Jan-16 | 1.129 | 1.032 | 1.235 |
| Jan-15 / Jan-16 | 1.007 | 0.920 | 1.102 |
| Empty / Pair | 0.629 | 0.467 | 0.848 |
| Empty / Group | 0.505 | 0.371 | 0.689 |
| Pair / Group | 0.803 | 0.607 | 1.063 |

Tests were conducted on the log scale. Confidence intervals were back-transformed from the log scale.

**Table 3: Pairwise comparisons of interacting effects of survey time and transects with (Y) and without (N) groups on the mean number of empty corals per transect.**

| **Contrast** | **Ratio** | **Lower CI** | **Upper CI** |
| --- | --- | --- | --- |
| Feb-14,N / Aug-14,N | 1.277 | 0.362 | 4.502 |
| Feb-14,N / Jan-15,N | 0.859 | 0.242 | 3.049 |
| Feb-14,N / Jan-16,N | 1.376 | 0.385 | 4.910 |
| Feb-14,N / Feb-14,Y | 0.920 | 0.168 | 5.040 |
| Feb-14,N / Aug-14,Y | 1.335 | 0.074 | 24.028 |
| Feb-14,N / Jan-15,Y | 1.114 | 0.072 | 17.314 |
| Feb-14,N / Jan-16,Y | 1.400 | 0.086 | 22.713 |
| Aug-14,N / Jan-15,N | 0.673 | 0.118 | 3.854 |
| Aug-14,N / Jan-16,N | 1.077 | 0.161 | 7.207 |
| Aug-14,N / Feb-14,Y | 0.721 | 0.092 | 5.654 |
| Aug-14,N / Aug-14,Y | 1.045 | 0.084 | 12.984 |
| Aug-14,N / Jan-15,Y | 0.872 | 0.045 | 16.946 |
| Aug-14,N / Jan-16,Y | 1.096 | 0.051 | 23.396 |
| Jan-15,N / Jan-16,N | 1.601 | 0.289 | 8.862 |
| Jan-15,N / Feb-14,Y | 1.071 | 0.121 | 9.454 |
| Jan-15,N / Aug-14,Y | 1.553 | 0.064 | 37.922 |
| Jan-15,N / Jan-15,Y | 1.296 | 0.110 | 15.203 |
| Jan-15,N / Jan-16,Y | 1.628 | 0.076 | 34.731 |
| Jan-16,N / Feb-14,Y | 0.669 | 0.073 | 6.109 |
| Jan-16,N / Aug-14,Y | 0.970 | 0.037 | 25.452 |
| Jan-16,N / Jan-15,Y | 0.810 | 0.038 | 17.077 |
| Jan-16,N / Jan-16,Y | 1.017 | 0.084 | 12.295 |
| Feb-14,Y / Aug-14,Y | 1.450 | 0.150 | 13.986 |
| Feb-14,Y / Jan-15,Y | 1.210 | 0.131 | 11.201 |
| Feb-14,Y / Jan-16,Y | 1.521 | 0.149 | 15.476 |
| Aug-14,Y / Jan-15,Y | 0.835 | 0.035 | 19.711 |
| Aug-14,Y / Jan-16,Y | 1.049 | 0.035 | 31.171 |
| Jan-15,Y / Jan-16,Y | 1.257 | 0.057 | 27.817 |

Tests were conducted on the log scale. Confidence intervals were back-transformed from the log scale.

**Table 4. Predicted probabilities of inhabitance**

| **Dataset** | **Mean Coral Diameter (cm)** | **Inhabitance** | **Predicted Probability** |
| --- | --- | --- | --- |
| Feb-14 | 5 | Empty | 0.339 |
| Aug-14 | 5 | Empty | 0.661 |
| Jan-15 | 5 | Empty | 0.702 |
| Jan-16 | 5 | Empty | 0.651 |
| Feb-14 | 10 | Empty | 0.205 |
| Aug-14 | 10 | Empty | 0.496 |
| Jan-15 | 10 | Empty | 0.541 |
| Jan-16 | 10 | Empty | 0.482 |
| Feb-14 | 15 | Empty | 0.113 |
| Aug-14 | 15 | Empty | 0.329 |
| Jan-15 | 15 | Empty | 0.368 |
| Jan-16 | 15 | Empty | 0.314 |
| Feb-14 | 20 | Empty | 0.059 |
| Aug-14 | 20 | Empty | 0.195 |
| Jan-15 | 20 | Empty | 0.221 |
| Jan-16 | 20 | Empty | 0.181 |
| Feb-14 | 25 | Empty | 0.029 |
| Aug-14 | 25 | Empty | 0.105 |
| Jan-15 | 25 | Empty | 0.119 |
| Jan-16 | 25 | Empty | 0.095 |
| Feb-14 | 30 | Empty | 0.014 |
| Aug-14 | 30 | Empty | 0.053 |
| Jan-15 | 30 | Empty | 0.060 |
| Jan-16 | 30 | Empty | 0.047 |
| Feb-14 | 35 | Empty | 0.006 |
| Aug-14 | 35 | Empty | 0.025 |
| Jan-15 | 35 | Empty | 0.028 |
| Jan-16 | 35 | Empty | 0.022 |
| Feb-14 | 40 | Empty | 0.003 |
| Aug-14 | 40 | Empty | 0.012 |
| Jan-15 | 40 | Empty | 0.013 |
| Jan-16 | 40 | Empty | 0.010 |
| Feb-14 | 45 | Empty | 0.001 |
| Aug-14 | 45 | Empty | 0.005 |
| Jan-15 | 45 | Empty | 0.006 |
| Jan-16 | 45 | Empty | 0.004 |
| Feb-14 | 50 | Empty | 0.001 |
| Aug-14 | 50 | Empty | 0.002 |
| Jan-15 | 50 | Empty | 0.002 |
| Jan-16 | 50 | Empty | 0.002 |
| Feb-14 | 55 | Empty | 0.000 |
| Aug-14 | 55 | Empty | 0.001 |
| Jan-15 | 55 | Empty | 0.001 |
| Jan-16 | 55 | Empty | 0.001 |
| Feb-14 | 60 | Empty | 0.000 |
| Aug-14 | 60 | Empty | 0.000 |
| Jan-15 | 60 | Empty | 0.000 |
| Jan-16 | 60 | Empty | 0.000 |
| Feb-14 | 5 | Pair | 0.582 |
| Aug-14 | 5 | Pair | 0.304 |
| Jan-15 | 5 | Pair | 0.261 |
| Jan-16 | 5 | Pair | 0.300 |
| Feb-14 | 10 | Pair | 0.672 |
| Aug-14 | 10 | Pair | 0.436 |
| Jan-15 | 10 | Pair | 0.384 |
| Jan-16 | 10 | Pair | 0.425 |
| Feb-14 | 15 | Pair | 0.711 |
| Aug-14 | 15 | Pair | 0.553 |
| Jan-15 | 15 | Pair | 0.500 |
| Jan-16 | 15 | Pair | 0.529 |
| Feb-14 | 20 | Pair | 0.705 |
| Aug-14 | 20 | Pair | 0.625 |
| Jan-15 | 20 | Pair | 0.574 |
| Jan-16 | 20 | Pair | 0.584 |
| Feb-14 | 25 | Pair | 0.668 |
| Aug-14 | 25 | Pair | 0.644 |
| Jan-15 | 25 | Pair | 0.593 |
| Jan-16 | 25 | Pair | 0.586 |
| Feb-14 | 30 | Pair | 0.611 |
| Aug-14 | 30 | Pair | 0.620 |
| Jan-15 | 30 | Pair | 0.568 |
| Jan-16 | 30 | Pair | 0.549 |
| Feb-14 | 35 | Pair | 0.543 |
| Aug-14 | 35 | Pair | 0.569 |
| Jan-15 | 35 | Pair | 0.515 |
| Jan-16 | 35 | Pair | 0.490 |
| Feb-14 | 40 | Pair | 0.470 |
| Aug-14 | 40 | Pair | 0.503 |
| Jan-15 | 40 | Pair | 0.448 |
| Jan-16 | 40 | Pair | 0.422 |
| Feb-14 | 45 | Pair | 0.396 |
| Aug-14 | 45 | Pair | 0.431 |
| Jan-15 | 45 | Pair | 0.379 |
| Jan-16 | 45 | Pair | 0.353 |
| Feb-14 | 50 | Pair | 0.327 |
| Aug-14 | 50 | Pair | 0.360 |
| Jan-15 | 50 | Pair | 0.312 |
| Jan-16 | 50 | Pair | 0.288 |
| Feb-14 | 55 | Pair | 0.264 |
| Aug-14 | 55 | Pair | 0.294 |
| Jan-15 | 55 | Pair | 0.251 |
| Jan-16 | 55 | Pair | 0.231 |
| Feb-14 | 60 | Pair | 0.210 |
| Aug-14 | 60 | Pair | 0.236 |
| Jan-15 | 60 | Pair | 0.199 |
| Jan-16 | 60 | Pair | 0.181 |
| Feb-14 | 5 | Group | 0.079 |
| Aug-14 | 5 | Group | 0.035 |
| Jan-15 | 5 | Group | 0.038 |
| Jan-16 | 5 | Group | 0.049 |
| Feb-14 | 10 | Group | 0.123 |
| Aug-14 | 10 | Group | 0.069 |
| Jan-15 | 10 | Group | 0.075 |
| Jan-16 | 10 | Group | 0.093 |
| Feb-14 | 15 | Group | 0.176 |
| Aug-14 | 15 | Group | 0.118 |
| Jan-15 | 15 | Group | 0.132 |
| Jan-16 | 15 | Group | 0.157 |
| Feb-14 | 20 | Group | 0.236 |
| Aug-14 | 20 | Group | 0.180 |
| Jan-15 | 20 | Group | 0.206 |
| Jan-16 | 20 | Group | 0.235 |
| Feb-14 | 25 | Group | 0.303 |
| Aug-14 | 25 | Group | 0.251 |
| Jan-15 | 25 | Group | 0.288 |
| Jan-16 | 25 | Group | 0.319 |
| Feb-14 | 30 | Group | 0.375 |
| Aug-14 | 30 | Group | 0.327 |
| Jan-15 | 30 | Group | 0.373 |
| Jan-16 | 30 | Group | 0.404 |
| Feb-14 | 35 | Group | 0.451 |
| Aug-14 | 35 | Group | 0.406 |
| Jan-15 | 35 | Group | 0.457 |
| Jan-16 | 35 | Group | 0.488 |
| Feb-14 | 40 | Group | 0.527 |
| Aug-14 | 40 | Group | 0.486 |
| Jan-15 | 40 | Group | 0.539 |
| Jan-16 | 40 | Group | 0.568 |
| Feb-14 | 45 | Group | 0.602 |
| Aug-14 | 45 | Group | 0.564 |
| Jan-15 | 45 | Group | 0.616 |
| Jan-16 | 45 | Group | 0.643 |
| Feb-14 | 50 | Group | 0.672 |
| Aug-14 | 50 | Group | 0.637 |
| Jan-15 | 50 | Group | 0.686 |
| Jan-16 | 50 | Group | 0.710 |
| Feb-14 | 55 | Group | 0.735 |
| Aug-14 | 55 | Group | 0.705 |
| Jan-15 | 55 | Group | 0.748 |
| Jan-16 | 55 | Group | 0.769 |
| Feb-14 | 60 | Group | 0.790 |
| Aug-14 | 60 | Group | 0.764 |
| Jan-15 | 60 | Group | 0.801 |
| Jan-16 | 60 | Group | 0.818 |

Probability that corals of varying mean diameter would remain empty or be inhabited by either pair- or group-forming species of *Gobiodon*. Probabilities were predicted from a multinomial model performed in R using the nnet package [2].

**References**

1. Lenth R. Emmeans: Estimated marginal means, aka least-squares means. R package. 1.1.2 ed2018.

2. Venables W, Ripley B. Modern applied statistics with S. 4th ed: New York: Springer; 2002.
