# Supplementary material for: Repeated cyclone events reveal potential causes of sociality in coral-dwelling Gobiodon fishes
Source: PLoS One. 2018 Sep 5;13(9):e0202407. doi: 10.1371/journal.pone.0202407 (PMC6124712; doi:10.1371/journal.pone.0202407)
Supplement: S3 Table — Odds and associated confidence interval (CI) for each model coefficient. Vacant corals were the reference group. Odds = 1 indicate an equal chance that the coral would remain vacant or be inhabited by either a pair- or group-forming species. Odds > 1 indicate a greater chance of the coral being inhabited by either pair- or group-forming species rather than remaining vacant. Odds < 1 indicates a greater chance of the coral remaining vacant. (DOCX) [file pone.0202407.s006.docx]

**S3 Table: Model coefficients for the multinomial probability of occupancy.**

|  | **Intercept** | | | **Aug-14** | | | **Jan-15** | | | **Jan-16** | | |
| --- | --- | --- | --- | --- | --- | --- | --- | --- | --- | --- | --- | --- |
|  | **Odds** | **Lower CI** | **Upper CI** | **Odds** | **Lower CI** | **Upper CI** | **Odds** | **Lower CI** | **Upper CI** | **Odds** | **Lower CI** | **Upper CI** |
| **Pair** | 1.680 | 0.000 | 1.038 | 0.163 | 0.000 | -3.624 | 0.084 | 0.000 | -4.960 | 0.158 | 0.000 | -3.694 |
| **Group** | 0.099 | 0.000 | -4.625 | 0.221 | 0.000 | -3.018 | 0.135 | 0.000 | -3.999 | 0.483 | 0.000 | -1.454 |
|  | **Avg.Diam** | | | **Aug-14:Avg.Diam** | | | **Jan-15:Avg.Diam** | | | **Jan-16:Avg.Diam** | | |
|  | **Odds** | **Lower CI** | **Upper CI** | **Odds** | **Lower CI** | **Upper CI** | **Odds** | **Lower CI** | **Upper CI** | **Odds** | **Lower CI** | **Upper CI** |
| **Pair** | 1.103 | 0.000 | 0.197 | 1.023 | 0.000 | 0.046 | 1.056 | 0.000 | 0.110 | 1.023 | 0.000 | 0.045 |
| **Group** | 1.196 | 0.000 | 0.357 | 1.006 | 0.000 | 0.013 | 1.041 | 0.000 | 0.079 | 0.983 | 0.000 | -0.035 |

Odds and associated confidence interval (CI) for each model coefficient. Vacant corals were the reference group. Odds = 1 indicate an equal chance that the coral would remain vacant or be inhabited by either a pair- or group-forming species. Odds > 1 indicate a greater chance of the coral being inhabited by either pair- or group-forming species rather than remaining vacant. Odds < 1 indicates a greater chance of the coral remaining vacant.
